# Adult-Onset Still’s Disease (AOSD) Complicated by Haemophagocytic Lymphohistiocytosis (HLH) and Transient Thyrotoxicosis: A Diagnostic Challenge

**DOI:** 10.7759/cureus.95181

**Published:** 2025-10-22

**Authors:** Hana Sultan, Nishchil Patel

**Affiliations:** 1 Department of Internal Medicine, University Hospitals Plymouth NHS Trust, Plymouth, GBR; 2 Department of Endocrinology, University Hospitals Plymouth NHS Trust, Plymouth, GBR

**Keywords:** adult-onset still’s disease (aosd), autoimmune thyrotoxicosis, haemophagocytic lymphohistiocytosis (hlh), high ferritin, thyroiditis

## Abstract

Adult-onset Still’s disease (AOSD) is a rare systemic autoinflammatory disorder that can mimic infectious, autoimmune, and endocrine conditions. Overlapping features may delay diagnosis, as in this case, which was further confounded by abnormal thyroid function tests.

A 20-year-old woman presented with persistent fever, migratory arthralgia, myalgia, and a widespread urticarial rash following a sore throat. Initial work-up suggested thyrotoxicosis with weakly positive anti-TSH receptor antibodies, leading to treatment for presumed Graves’ disease. Despite therapy, she experienced recurrent admissions with worsening rash, polyarthritis, cytopenia, and seizures. Serial investigations revealed markedly elevated inflammatory markers and ferritin, evolving splenomegaly, and biochemical features of systemic inflammation. Autoimmune and infectious screens were negative. She fulfilled the criteria for AOSD complicated by haemophagocytic lymphohistiocytosis (HLH). Treatment with anakinra and dexamethasone achieved marked clinical improvement, with subsequent normalization of hematological and thyroid profiles.

AOSD with HLH is a life-threatening overlap syndrome requiring urgent recognition and treatment. Markedly elevated ferritin levels and multi-system involvement are pivotal diagnostic clues. Thyroid dysfunction, including transient thyroiditis, may occur in systemic inflammatory states, potentially delaying diagnosis.

This case highlights the importance of systematic re-evaluation when clinical and biochemical findings diverge from a presumed single-organ pathology. Early multidisciplinary involvement and targeted therapy can be lifesaving in AOSD-HLH.

## Introduction

We report a complex case of adult-onset Still’s disease (AOSD) with secondary hemophagocytic lymphohistiocytosis (HLH), initially mimicking Graves’ disease and thyrotoxicosis. AOSD is a rare systemic autoinflammatory disorder characterized by spiking fever, evanescent rash, arthritis, and elevated inflammatory markers [1,2]. The incidence rate is approximately 0.16-0.4 per 100,000 people [2]. Diagnosis is challenging and often requires the exclusion of infectious, malignant, and autoimmune conditions with similar presenting features [1,2]. HLH is a severe, life-threatening syndrome characterized by excessive immune activation, resulting in a hyperinflammatory state that can cause tissue damage, multiorgan failure, and, if untreated, death. HLH is classified into primary (familial) and secondary (acquired) forms and is usually seen in children. The reported incidence in the adult population is approximately 1 in 2,000 [3].

An overactive thyroid state can cause symptoms such as weight loss, anxiety, tremors, palpitations, and heat intolerance. Thyroid dysfunction in systemic inflammatory diseases, usually due to thyroiditis, can at times obscure the clinical picture. This case illustrates the diagnostic challenges posed by overlapping endocrine and autoinflammatory features [4].

## Case presentation

Initial presentation

A 20-year-old healthcare assistant presented with a two-month history of persistent fever, migratory arthralgia, myalgia, and a widespread urticarial rash worsened by sun exposure. Symptoms began after a sore throat treated with amoxicillin, followed by a generalized rash within 24 hours. She had no significant past medical history, family history of autoimmune disease, or history of smoking, alcohol, or recreational drug use.

On examination, she was febrile (>39°C) with a widespread urticarial rash, mild pharyngitis, generalized lymphadenopathy, and migratory polyarthritis. Initial laboratory tests suggested thyrotoxicosis (suppressed TSH, mildly raised free T3 and T4, weakly positive anti-TSH receptor antibodies) and elevated inflammatory markers (CRP 180 mg/L, ferritin 475 μg/L) (Table 1).

**Table 1:** Routine blood tests during first admission. Hb: Hemoglobin; WCC: White cell count; eGFR: Estimated glomerular filtration rate; LFTs: Liver function tests; TSH: Thyroid-stimulating hormone; Free T3: Free triiodothyronine; Free T4: Free thyroxine; TSH: Thyroid-stimulating hormone.

Marker	Value	Reference range
CRP	180	0.1-5mg/L
Hb	108	120-155g/L
WCC	9.3	3.6-9.2x10^9^/L
eGFR	>90	90-120 mL/min/1.73 m^2^
Ferritin	475	30-200ug/L
Liver function tests (LFT)	Normal	
TSH	<0.004	0.35-4.94 mIU/L
Free T3	7.3	2.9-4.9 pmol/L
Free T4	25.3	9-19 pmol/L
Anti-TSH receptor antibodies	2.14	0-2 IU/L

Blood and urine cultures returned negative. She was treated for presumed idiopathic urticaria and Graves’ disease with fexofenadine, montelukast, carbimazole, and propranolol.

Subsequent admissions

Over the following weeks, she was readmitted with worsening rash, persistent fevers, polyarthritis, and anemia (Table 2). Autoimmune screens and infectious serology (HIV, hepatitis B/C, syphilis, Lyme disease) were negative (Table 3). Imaging showed wrist synovitis and, later, splenomegaly with ascites and features suggestive of developing hepatitis, though liver biochemistry remained normal.

**Table 2:** Routine blood tests during second admission. Hb: Hemoglobin; WCC: White cell count; eGFR: Estimated glomerular filtration rate; LFTs: Liver function tests; TSH: Thyroid-stimulating hormone; Free T3: Free triiodothyronine; Free T4: Free thyroxine; TSH: Thyroid-stimulating hormone receptor.

Marker	Value	Reference Range
CRP	320	0.1-5 mg/L
Hb	91	120-155 g/L
WCC	14.2	3.6-9.2 × 10⁹/L
eGFR	>90	90-120 mL/min/1.73 m²
Ferritin	2,475	30-200 µg/L
Thyroid function tests		
TSH	<0.004	0.35-4.94 mIU/L
Free T₃	6.3	2.9-4.9 pmol/L
Free T₄	25	9-19 pmol/L
Anti-TSH receptor antibodies	0.56	0-2 IU/L
Liver function tests (LFTs)	Normal	-

**Table 3:** Immunology tests and viral markers.

Marker	Value	Reference Range
Anticardiolipin IgG antibody	20	0-9.9 U/mL
Anticardiolipin IgM antibody	Negative	-
IgG β₂-glycoprotein antibody	113	0-10 U/mL
IgM β₂-glycoprotein antibody	3	0-10 U/mL
ANA screen	Positive	-
Anti-dsDNA antibody	Negative	-
Anti-tTG antibody	Negative	-
Epstein-Barr virus serology	Negative	-
Syphilis - TP antibody	Negative	-
Lyme disease serology	Negative	-
Anti-streptolysin O antibody	Negative	-
Lupus anticoagulant screen	Negative	-
ENA screen	Negative	-
Complement C3	1.86	0.83-1.30 g/L
Complement C4	0.27	0.15-0.57 g/L
Rheumatoid factor	Negative	-
Hepatitis B surface antigen (HBsAg)	Negative	-
Hepatitis C antibody screen	Negative	-
HIV screen	Negative	-
Hepatitis B core antibody (anti-HBc)	Negative	-

She was managed symptomatically and discharged once clinically better, with a plan for outpatient follow-up.

However, she was admitted a third time shortly after discharge, following generalized seizures witnessed at home by her parents, who described her eyes rolling back, followed by a full tonic-clonic seizure lasting approximately 2-3 minutes. There was no incontinence or tongue biting during the episode. Post-seizure, she was confused and fatigued, with a post-ictal period lasting around 5-10 minutes. Seizure-like activity was confirmed on electroencephalogram (EEG). MRI of the brain revealed a Chiari I malformation, which was deemed incidental. Her previous symptoms persisted, and this time her ferritin was markedly elevated (22,069 μg/L).

In light of negative tests for other conditions, alongside a progressively rising ferritin since first presentation (475 → 2475 → 22,069 μg/L) and now marked elevation, she was diagnosed with HLH.

Diagnosis and management

Following multidisciplinary review, she fulfilled the criteria for AOSD (Yamaguchi criteria) and secondary hemophagocytic lymphohistiocytosis (HLH-2004 criteria). Carbimazole was discontinued due to possible drug-induced agranulocytosis. Anakinra 100 mg twice daily and dexamethasone 14 mg daily were initiated, alongside seizure prophylaxis and antimicrobial prophylaxis.

Genetic testing excluded familial HLH. She demonstrated rapid improvement with resolution of fevers, rash, cytopenia, and normalization of inflammatory markers. Dexamethasone was tapered and stopped over the next few weeks after the initial inflammation had settled. Thyroid function normalized completely over the next few months without further thyroid-specific treatment, consistent with transient thyroiditis secondary to systemic inflammation (Table 4).

**Table 4:** Summary of thyroid tests over time. TSH: Thyroid-stimulating hormone; Free T3: Free triiodothyronine; Free T4: Free thyroxine.

Thyroid function tests	Value						Reference range
	First admission	Second admission	Third admission	Endocrine OP review (1 month post discharge of 3rd admission)	Follow up bloods	Endocrine OP review euthyroid (7 months post discharge since 4th admission)	
TSH	<0.004	<0.004	<0.004	0.014	0.14	0.59	0.35-4.94 miu/L
Free T3	7.3	6.3	2.6	1.7	1.9	4	2.9-4.9 pmol/L
T4	25.3	25	12.4	8.3	<5.2	14.8	9-19 pmol/L
Anti-TSH receptor antibodies	2.14		2.19	0.56		0.53	0-2 IU/L

She remained seizure-free at follow-up. Prophylactic anti-epileptic treatment was continued, with a plan to stop if she remained seizure-free for at least two years and following a repeat EEG at that time. She remains on anakinra on alternate days and methotrexate 20 mg once weekly.

## Discussion

AOSD remains a challenging diagnosis due to its heterogeneous presentation and the necessity of excluding multiple differential diagnoses [1,2,5]. The classic triad of spiking fevers, evanescent rash, and arthritis may be incomplete early in the disease course [2].

Transient thyrotoxicosis in systemic inflammatory diseases is thought to result from cytokine-mediated thyroiditis rather than primary autoimmune thyroid disease [6]. IL-1, IL-6, and TNF-α have been implicated in the pathogenesis of both AOSD and destructive thyroiditis [1,5]. Previous reports of AOSD-associated painless thyroiditis support our observation that thyroid abnormalities may resolve following control of systemic inflammation [6]. Our patient fulfilled the criteria of fever, associated rash, sore throat, and leukocytosis, alongside negative cultures that ruled out infection and a negative screen for rheumatological causes.

HLH is a hyperinflammatory syndrome that can complicate AOSD, with potentially fatal consequences if not promptly recognized [4,7]. Our patient met the HLH-2004 diagnostic criteria, including fever, cytopenia, hyperferritinemia, splenomegaly, and elevated soluble IL-2 receptor levels [4]. Ferritin levels >10,000 μg/L, as seen here, are highly suggestive of HLH in the appropriate clinical context [4].

Management requires the urgent initiation of immunosuppressive therapy. Anakinra, an IL-1 receptor antagonist, has emerged as an effective first-line option for both refractory AOSD and AOSD-associated HLH [7]. The rapid resolution of our patient’s symptoms after Anakinra initiation underscores its role in modulating cytokine-driven inflammation [2,7]. Close multidisciplinary collaboration between rheumatology, hematology, neurology, and endocrinology was essential for a successful outcome.

## Conclusions

This case demonstrates how endocrine abnormalities, particularly thyroid dysfunction, can confound the clinical recognition of systemic autoinflammatory diseases such as AOSD. Markedly elevated ferritin levels and multi-system involvement should prompt consideration of HLH and trigger urgent rheumatological input. In AOSD, despite life-threatening complications, the prognosis remains good and mortality rates are low, whereas HLH can often be fatal. Early recognition and initiation of treatment are crucial in reducing mortality. Multidisciplinary care and early targeted biologic therapy can be lifesaving.

Clinicians should therefore consider AOSD in patients presenting with persistent fever, rash, and systemic inflammation when common infectious and autoimmune causes have been excluded. Markedly elevated ferritin serves as a key diagnostic clue for both HLH and AOSD and should prompt urgent evaluation. Thyroid function abnormalities observed in acute systemic illness may often reflect transient thyroiditis rather than primary thyroid disease.
